# Parkinson’s Disease Dementia and Dementia with Lewy Bodies Have Similar Neuropsychological Profiles

**DOI:** 10.3389/fneur.2018.00123

**Published:** 2018-03-12

**Authors:** Georgina M. Aldridge, Allison Birnschein, Natalie L. Denburg, Nandakumar S. Narayanan

**Affiliations:** ^1^Department of Neurology, University of Iowa, Iowa City, IA, United States; ^2^Division of Behavioral Medicine and Clinical Psychology, Cincinnati Children’s Hospital Medical Center, Cincinnati, OH, United States

**Keywords:** parkinsonism, diagnosis, Lewy bodies, dementia, synuclein, Parkinson’s disease dementia, dementia with Lewy bodies, cognitive

## Abstract

Parkinson’s disease dementia (PDD) and dementia with Lewy bodies (DLB) are common causes of dementia worldwide. Although considered separate entities based on the relative temporal onset of motor symptoms vs. diagnosis of dementia, it is unknown if these diseases truly have distinct cognitive profiles. We hypothesized that patients divided into PDD and DLB categories strictly by temporal criteria would have different neuropsychological profiles. We investigated this question *via* neuropsychological testing of PDD and DLB patients at the University of Iowa. We performed retrospective chart analysis and review of neuropsychological testing of clinically diagnosed patients with PDD or DLB, who had presented to University of Iowa’s dementia and movement disorder clinics. Forty-seven patients diagnosed by the treating neurologist as PDD or DLB were included. Neuropsychological performance was compared between groups, and as a function of the relative timing of the motor diagnosis vs. diagnosis of dementia. We found that both PDD and DLB patients showed severe deficits in executive function, visual–spatial processing, and verbal learning. However, we found no significant differences in neuropsychological performance between groups, and neuropsychological performance could not reliably account for the relative timing of motor diagnosis vs. diagnosis of dementia. Our data support the idea that DLB and PDD are on a neuropsychological spectrum.

## Introduction

Together, Parkinson’s disease dementia (PDD) and dementia with Lewy bodies (DLB) represent the second most common cause of dementia worldwide (1). Both diseases are synucleinopathies and involve pathological accumulations of the protein alpha-synuclein (2). The cognitive deficits in PDD and DLB are distinct from Alzheimer’s dementia, with notable relative preservation of delayed memory, but severely impaired executive function, attention, and visuospatial skills (3–5). Parkinson’s disease is a disorder of motor symptoms, but approximately 30% of such patients have cognitive symptoms at initial diagnosis and as many as 80% will develop cognitive symptoms at some point in their disease (6, 7). In DLB, cognitive symptoms appear before, or at the same time, as motor symptoms (2).

Core diagnostic features of DLB include parkinsonism, visual hallucinations, fluctuations, and rapid eye movement (REM) sleep behavior disorder. These features are also common in PDD, but for this diagnosis, patients need a prior diagnosis of Parkinson’s disease (2). Crucially, if a patient has parkinsonism, PDD vs. DLB can only be clinically distinguished by the relative temporal onset of cognitive vs. motor disease. According to criteria set forth by the consensus report of the Lewy Body Consortium (2), PDD is diagnosed when there is at least a 1-year interval between onset of parkinsonian motor symptoms and diagnosis of dementia. Many clinical studies use more stringent criteria of temporal onset of motor symptoms vs. dementia to separate DLB from PDD, thus the full spectrum of these diseases has rarely been studied. Accordingly, it is unclear if PDD and DLB are really two different diseases affecting distinct cortical circuits and thus separate neuropsychological profiles, or a spectrum of one disease (8).

We investigated this issue by comparing the neuropsychological profiles of patients diagnosed with PDD or DLB at our clinical center at the University of Iowa. We conducted a retrospective chart analysis of 47 patients with an existing diagnosis of either PDD or DLB based on consensus criteria, who had formal neuropsychological testing as part of their standard workup. We defined PDD or DLB based solely on temporal profiles of date of diagnosis of parkinsonism vs. dementia. If these diseases had distinct neuropsychological profiles: (a) they should have distinct performance on neuropsychological tests and (b) the relative timing of motor diagnosis vs. dementia diagnosis should predict neuropsychological function.

## Materials and Methods

### Selection of Patients

Patients who were seen in the Benton Neuropsychology Laboratory, Department of Neurology, University of Iowa Hospitals and Clinics, were screened for use in a retrospective chart review using the diagnoses of PDD or DLB. This study was carried out in accordance with the recommendations of the University of Iowa Institutional Review Board. Initial patient charts were gathered using two methods: (1) all neuropsychological charts from 2005 to 2015 were scanned by keyword search for previously assigned labels (*PDD, DLB, Parkinson’s disease with dementia, dementia with Lewy bodies*, and *Lewy body dementi*a) and (2) all charts from patients presenting to movement disorder clinic during a 1-year interval during 2014–2015 were screened for prior neuropsychological testing, and if present, evidence of the treating physicians diagnosis of either PDD or DLB. Three hundred and ten charts were found with these methods and preliminarily screened for inclusion criteria (diagnosis of DLB or PDD, as well as diagnosis of dementia by neuropsychological testing).

Eighty-two patient charts that had a diagnosis of PDD or DLB were reviewed together by a consensus panel of two neurologists and a neuropsychologist. Patients were included only if they had been diagnosed by the treating neurologist as either PDD or DLB. Patients were excluded if they had evidence of comorbid neurological disease that could potentially explain the dementing illness, including, but not limited to significant vascular disease, traumatic brain injury, or Alzheimer’s disease. Patients were also excluded if progressive supranuclear palsy, multiple system atrophy, or other causes of parkinsonism was the most likely diagnosis. 47 patients remained after exclusion criteria were applied.

Neuropsychological evaluation was used for dating the diagnosis of dementia unless prior evidence in the chart suggested a clear earlier date; typically, neuropsychological testing occurred within a few months of clinical referral. All patients had been evaluated by a neurologist, and their neurological evaluation was used for parkinsonism diagnosis. Patient follow-up was determined by the treating physician. Patients who did not have a diagnosis of dementia or any cognitive complaints were excluded from analysis.

Patients were then retrospectively diagnosed with either PDD or DLB according to the temporal criteria outlined by the DLB consortium (2), regardless of other features (Table 1). We determined the dates when parkinsonism and dementia were diagnosed by the treating physician or neuropsychologist. The difference (dementia diagnosis date − motor diagnosis date) was considered the cognitive-motor interval (CMI). Patients with a CMI of 1 year or less were diagnosed as DLB based on the 1-year rule recommended for research by the DLB consortium (Table 1). For patients without evidence of parkinsonism, the date of diagnosis of DLB by other core symptoms was used (two patients). Of note, these estimates were calculated without considering the neuropsychological profile of each patient.

**Table 1:** Demographics.

Diagnosis		Number	Age	Education	% Right handed	% Male	% Depression	Average CMI (years)
Dementia with Lewy bodies	Mean	22	72.3	12.1	72.7	77.3	50.0	0.09 (−1.5 to 1)
	SD		6.6	2.7				0.62

Parkinson’s disease dementia	Mean	25	71.5	14.1	84.0	64.0	45.5	7.9 (1.06 to 23)
	SD		6.0	3.0				6.5

	*p*-Value		0.67	0.02, 0.10^a^	0.28^b^	0.32^b^	0.87^b^	

*Continuous variables were compared by Student’s t-test*.

*There was a trend towards increased education in Parkinson’s dementia group, but this did not survive correction for multiple comparisons using a false-discovery-rate approach^a^. Categorical variables were compared by Pearson chi-square^b^. Patients with diagnosis of parkinsonism more than 1.0 years before diagnosis of dementia [Cognitive-Motor Interval (CMI) > 1] were classified as Parkinson’s disease dementia*.

### Neuropsychological Measures

Multiple neuropsychological measures were analyzed to assess patients’ broad cognitive functioning. Because data were collected retrospectively from a clinical referral, not all participants received each of the possible neuropsychological tests. Tests were only included if they had been administered to at least 60% of patients. Thus, we included data from the following neuropsychological tests.

#### Attention and Concentration

Measures that test attention and concentration in our study include the Digit Span subtest and the Arithmetic subtest from the Wechsler Adult Intelligence Scale-Third Edition (and other versions) [WAIS-III (9)]; and the Benton Visual Retention Test [BVRT (10)]. For the WAIS-III Digit Span test, participants are required to repeat a string of digits either forwards (attention) or backwards (concentration). A combined score was used for analysis. During the BVRT, subjects are presented with 10 different designs, each containing one or more figures. Each design is exposed for 10 s, followed by immediate reproduction from memory by the participant. During arithmetic, patients are presented with a series of verbal reasoning/math problems and asked to produce a numerical response without the use of paper and pencil.

#### Orientation and Anterograde Memory

Orientation to time was assessed using several questions from the Benton Orientation Task (11, 12). As the Orientation task produces non-normal data and does not have associated age-adjusted norms, patients were either coded as oriented to time (score > −2), or non-oriented. The Rey Auditory Verbal Learning Test [AVLT (13)] and Logical Memory subtest of the WMS-R (14) were used to measure the patient’s anterograde verbal memory for word lists and context-associated memory, respectively. For the AVLT, a list of 15 words was read to the participant on five immediate consecutive trials. After each trial, the participant recalled as many words as possible (immediate) and then again 30 min later (delay). The Logical Memory task involves recollection of a verbally presented story both immediately (Logical Memory 1) and after a 30-min delay (Logical Memory 2). Finally, the delay condition of the Rey–Osterrieth Complex Figure Test was used to measure anterograde non-verbal memory by presenting a complex figure and asking the participant to copy the figure (Rey–Osterrieth Copy) and then reproduce the figure after a 30-min delay (Rey–Osterrieth Delay) (15).

#### Language

Language was assessed with the Boston Naming Test [BNT (16)] and the Controlled Oral Word Association Test (COWAT/phonemic fluency) (11, 12). The BNT was utilized to assess confrontation naming abilities using up to 60 different line drawings. The COWAT assessed a participant’s phonemic verbal fluency by quantifying the number of C, F, and L words the patient could list, each in 1 min.

#### Visuospatial

Visuospatial abilities were measured with the Block Design subtest of the WAIS-III (9) and the Rey–Osterrieth Complex Figure Test-Copy Condition (discussed earlier). The WAIS-III Block Design measured three-dimensional visuoconstruction as the participant was asked to construct a pictured design using a set of 4 or 9 identical blocks.

#### Psychomotor

To assess visuomotor sequencing, Trail Making Test-Part A (17) was utilized. Participants were timed connecting consecutively numbered circles from 1 to 25 as quickly as possible. The WAIS-III Digit Symbol/Coding subtest (9) was also used to measure clerical speed and accuracy; subjects were presented with a symbol-number key and asked to insert the appropriate symbol in as many numbered boxes as possible within 120 s.

#### Executive Functioning

Patient’s executive functions were measured using the Trail Making Test-Part B (17). This test measures visuomotor set-shifting as participants were asked to connect consecutively numbered and lettered circles, alternating number with letter, as quickly as they can (time in seconds). The WAIS-III Similarities (9) were used to measure verbal abstraction as the participant was asked to explain how a pair of words were alike or similar.

#### Mood

The Beck Depression Inventory (18) or the Geriatric Depression Scale (19) was used to gage evidence of depression. To combine data between tests, patients were coded into three categories: mild to no depressive symptoms, moderate depression, and severe depression.

#### Core Features

Each patient’s chart was examined for evidence of visual hallucinations, reported symptoms of REM sleep behavior disorder (acting out dreams, falling out of bed), and reported symptoms of cognitive fluctuation. Patients who lacked documentation (0 for hallucinations, 3 for REM, and 11 for fluctuations) were not included in analysis of that feature.

### Statistics

We analyzed our data in two ways. First, we assigned a diagnosis of PDD or DLB to each patient using the temporal criteria and tested if neuropsychological measures differed between the two groups. Neuropsychological data were normalized to age-appropriate norms, and *z*-transformed to ensure comparable variance. The Mann–Whitney *U* (non-parametric) was used to test for differences between the groups, as several of the neuropsychological tests were non-normally distributed. *p*-Values were corrected for multiple comparisons using a false-discovery-rate approach; we present both values to aid in interpretation. A Bayes factor was calculated to express confidence in the null hypothesis. Second, we used a linear mixed effects model to account for variance in the timing of motor diagnosis vs. dementia on neuropsychological testing (lmer from the lme4 package in R). Because the CMI was non-normal, we used a Box–Cox transformation to convert it to a normalized distribution. To compare the two clinical samples, PDD and DLB, independent sample *t-*tests were conducted on age at time of neuropsychological testing and years of completed education. Chi-square analysis was used to analyze categorical measures, including sex, handedness, orientation, and depression.

## Results

**Figure 1:**
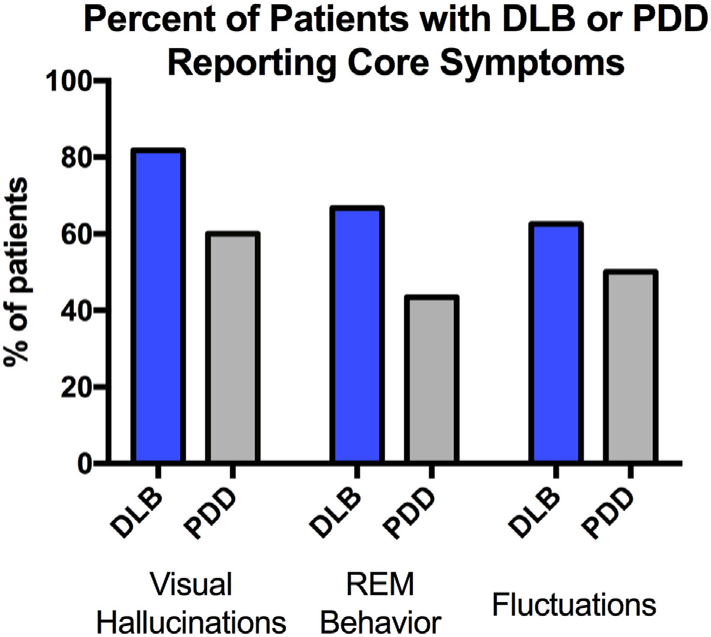
Core features were compared between dementia with Lewy bodies (DLB) and Parkinson’s disease dementia (PDD). Pearson’s chi-square analysis showed no significant difference between the groups in visual hallucinations/illusions (*p* = 0.10), symptoms of rapid eye movement (REM) sleep behavior disorder (*p* = 0.12), or evidence of fluctuations (*p* = 0.45).

According to DLB consortium criteria, there were 22 patients with DLB and 25 patients with PDD (2). Age, education, and demographic characteristics are represented in Table 1. The mean age of our patients was 71.5 ± 6.6 years for PDD vs. 72.3 ± 6.0 years for DLB. In our sample, PDD patients tended to be slightly more educated than DLB patients (*p* = 0.02, *p*_FDR_ = 0.19). There was a trend toward increased visual hallucinations/illusions in DLB (82% of DLB and 60% of PDD, *p* = 0.10). Cognitive fluctuations and symptoms of REM sleep behavior disorder were also more prevalent in DLB in our sample (Figure 1). However, in all cases, there was no statistical significance in core features between groups.

**Figure 2:**
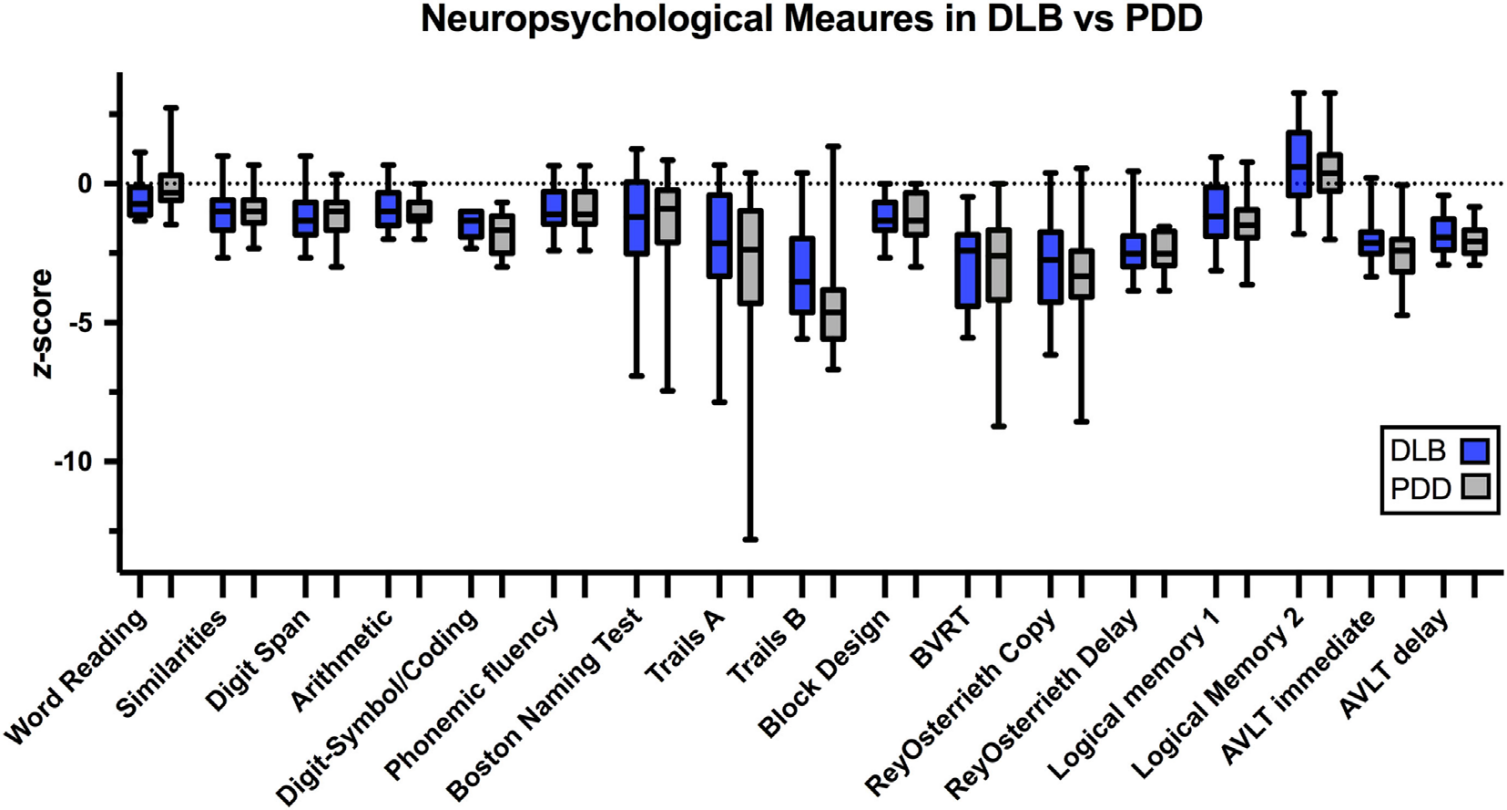
Box-and-whisker plots of DLB and PDD patients showing range of *z*-scores. Boxes represent *z*-scores from the 25th to 75th percentile. Whiskers represent the minimum and maximum *z*-scores. Abbreviations: DLB, Dementia with Lewy Bodies; PDD, Parkinson’s Disease Dementia; BVRT, Benton Visual Retention Test; AVLT, Rey Auditory Verbal Learning Test.

We found no reliable neuropsychological differences between PDD and DLB patients (Figure 2; Table 2). Notably, Trails B appeared different between groups on uncorrected hypothesis testing (*p* = 0.01), but this did not survive false-discovery-rate (FDR) correction (*p*_FDR_ = 0.18). One approach to quantify the evidence for a hypothesis is the Bayes Factor (20), which is derived from a calculation of the probabilities of the null hypothesis and an alternative. Values around 0.3 provide moderate evidence for the null hypothesis that there is no difference between PDD and DLB, whereas values >1 provide evidence to reject the null hypothesis. Most Bayes Factor differences between PDD and DLB were far below 1, with Trials B close to 1 (i.e., no evidence). Taken together, these data do not support the idea that PDD and DLB consistently differ in neuropsychological performance. Secondly, the critical feature differentiating DLB from PDD is the timing of motor vs. cognitive symptoms. To analyze if the temporal profile of symptom onset accounted for a significant degree of the variance in neuropsychological performance, we used linear mixed effects models controlling for age and education. Again, we could find no significant explanatory variables (Table 3), although there was a trend for Trails B (*p* = 0.02; *p*_FDR_ = 0.09). Of note, regression was only conducted on selected variables with the most explanatory power [Trails B, word reading, AVLT (delayed), Digit Span, and Logical Memory 1 (immediate)]; other tests explained minimal variance and were excluded. Taken together, these data suggest that neuropsychological performance was similar between DLB vs. PDD and was not explained by the relative timing of motor vs. cognitive diagnosis.

**Table 2:** Neuropsychological profile in dementia with Lewy bodies and Parkinson’s disease dementia (PDD).

Neuropsychological measure	Cognitive domain	Dementia with Lewy bodies			PDD			*p*-Value	Bayes
		Raw	SD	*z*-Score	Raw	SD	*z*-Score		
Oriented to time (% of patients)	Orientation/memory	50%			52%			0.89^a^	
Word reading	Premorbid education	90.87	9.8	−0.61	99.65	16.3	−0.02	0.07	1.02
Similarities	Abstraction	7.18	2.9	−0.94	6.86	2.4	−1.04	0.89	0.24
Digit Span (combined score)	Attention/conc.	6.52	2.8	−1.16	6.44	2.4	−1.19	0.92	0.22
Arithmetic	Concentration	7.53	2.5	−0.82	6.75	1.7	−1.08	0.47	0.40
Digit symbol/coding	Accuracy	5.56	1.6	−1.48	4.69	2.3	−1.77	0.31	0.50
Phonemic fluency (COWAT)	Language	23.24	11.5	−0.92	23.43	9.3	−0.95	0.91	0.31
Boston Naming Test	Language	49.24	7.6	−1.45	49.57	6.6	−1.37	0.90	0.24
Trails A (s)	Psychomotor speed	82.41	37.2	**−2.41**	91.88	44.1	**−3.21**	0.50	0.33
Trails B (s)	Executive function	283.23	88.1	**−3.28**	331.08	59.5	**−4.48**	0.01^b^	2.70
Block design	Visual–spatial/executive	6.06	2.0	−1.32	6.47	2.8	−1.18	0.44	0.28
Benton visual retention (errors)	Visual–spatial/attention	14.63	3.4	**−2.92**	15.231	4.2	**−3.29**	0.95	0.65
Rey–Osterrieth (copy)	Visual–spatial	16.55	5.0	**−3.03**	16.13	6.6	**−3.15**	0.71	0.23
Rey–Osterrieth (delay)	Delayed visual memory	5.81	4.1	**−2.42**	5.52	3.1	**−2.50**	0.88	0.23
Logical Memory 1 (immediate)	Immediate verbal memory	23.31	11.0	−1.03	21.55	8.4	−1.37	0.40	0.30
Logical Memory 2 (delay)	Delayed verbal memory	9.63	6.8	0.86	8.30	5.9	0.53	0.63	0.36
AVLT—immediate (trials 1–5)	Verbal learning	24.35	7.6	**−2.02**	21.37	7.6	**−2.38**	0.26	0.45
AVLT—delay	Delayed verbal memory	2.86	2.0	−1.86	2.42	1.7	−2.00	0.47	0.28

*Impairment was defined as *z*-score >2 SDs below the mean and are bolded/shadowed. Borderline scores (>1.33 below mean) are underlined. DLB and PDD groups were compared with Mann–Whitney *U* or, where noted^a^: Pearson chi-square. Uncorrected *p*-values are shown. Corrected *p*-value for Trails B^b^ was 0.18 with false-discovery-rate approach*.

*AVLT, Rey Auditory Verbal Learning Test; COWAT, Controlled Oral Word Association Test; DLB, dementia with Lewy bodies*.

**Table 3:** Estimates of regression coefficients and *p*-values for a linear mixed effects model of neuropsyschological tests vs. the time between motor diagnosis and cognitive diagnosis.

Test	Estimate	SE	*T*	*p*	FDR*p*
Trails B	−0.11	0.05	2.34	0.02	0.09
Word reading	−0.07	0.08	0.86	0.39	
AVLT (delay)	0.14	0.12	1.2	0.23	
Digit Span	0.7	0.1	0.74	0.46	
Logical Memory 1	0.03	0.08	0.46	0.64	

*p-Values <0.05 were corrected using false-discovery-rate (FDR) approach*.

*AVLT, Rey Auditory Verbal Learning Test*.

## Discussion

We investigated if PDD and DLB had distinct neuropsychological profiles. We compared neuropsychological performance between PDD and DLB and found few consistent differences. Furthermore, the key differentiating factor between PDD and DLB is the relative timing of motor onset of disease vs. dementia; we found that this variable also did not explain neuropsychological performance. These data provide evidence that PDD and DLB may be on a spectrum rather than two separate entities. Because these two conditions already share overlapping symptoms and pathophysiological features such as the aggregation of alpha-synuclein, these data provide further evidence that PDD and DLB may be clinical manifestations of the same disease.

Our study is distinct in using one key feature, the relative temporal onset of motor vs. cognitive symptoms, to compare neuropsychological data between PDD and DLB patients. While to the best of our knowledge, no other study has evaluated the correlation between CMI and neuropsychological profile, prior studies have attempted to determine differences in neuropsychological profile between groups divided by similar temporal rules. Despite strictly limiting PDD cases to those with long-standing Parkinson’s disease, most studies have not found reliable differences in neuropsychological function between PDD and DLB (3, 21, 22). A recent meta-analysis provided no statistical evidence for differences in neuropsychological profile between the groups (23). However, the topic still remains under debate as some studies have found differences. For instance, Aarsland et al. (5) found no differences between PDD and DLB on a dementia rating scale in measures of attention, initiation, construction or memory but did see a difference in a measure of conceptualization in the mild–moderate dementia subgroup. Filoteo et al. (24) found differences in verbal learning and memory, with DLB patients performing worse. However, only the DLB patients were diagnosed pathologically, and some were found to have concomitant Alzheimer’s disease pathology that may have contributed to their deficits. Furthermore, the majority of these pathologically defined DLB patients were initially diagnosed as Alzheimer’s disease, and therefore may represent a different population of Lewy body pathology. A small study by Mondon et al. (25) showed decreased performance in a DLB group on visual object recognition. The patients in the PDD group in this study were required to have parkinsonism for at least 6 years before diagnosis of dementia. Interestingly, a study by Janvin et al. (26) classified different cognitive profiles, such as subcortical and cortical, in a group of DLB/PDD patients. They found various cognitive profiles within each group, but the frequency of each was similar between PDD and DLB groups.

In our study, we found no significant differences between the groups. PDD patients had worse scores on Trails B, a measure of executive function, but the difference did not survive correction for multiple comparisons. While it is possible this difference might be significant with larger sample size, on average, both groups showed severe deficits in this area (>3 SD below mean), suggesting the overall cognitive profile is similar. Given the severity, it would be interesting to see if pathological data would better explain the variability in this test.

Patients with later cognitive onset (PDD patients) showed a trend toward greater education (total years) and higher scores on word reading (a measure of premorbid education). While our sample is not large enough to draw conclusions in this area, there is early evidence in the literature that cognitive reserve may be protective against dementia, potentially leading to delayed dementia diagnosis (27). This hypothesis warrants further evaluation in the future.

Taken together, these studies suggest that if there are different cognitive profiles within DLB/PDD, the current method of clinically dividing patients by temporal onset is not reflective of these differences. It is unclear if further mechanistic detail, such as measurements of cortical alpha-synuclein pathology, cortical function *via* EEG or fMRI, or neurotransmitter levels will contribute to separating PDD vs. DLB (28–31).

Beyond cognitive function, prior studies also have found no differences when comparing autonomic dysfunction (32), degree of parkinsonism (33) or neuroleptic sensitivity (34). Some studies have found distinctions in core features of the disease, such as greater visual hallucinations and fluctuations in attention in DLB (35, 36), and reduced levodopa responsivity (37). In each case, however, there is considerable overlap. For example, studies show 33–55% of DLB patients do display response to levodopa (37, 38), further complicating clinical efforts to separate PDD vs. DLB, and suggesting DLB patients should not be denied a trial of this medication.

Prior studies have shown hallucinations are more common in DLB compared with PDD patients (76 vs. 54%), when patients are divided based on consensus criteria and postmortem autopsy data (35). This is in line with our data showing evidence of hallucinations in 82 vs. 60% of patients with temporally defined DLB and PDD. Visual hallucinations may develop due to accumulation of Lewy pathology in the cortex and thus be more prevalent when cognitive symptoms occur earlier. Alternatively, visual hallucinations, being prominent and easy to identify, may lead to earlier diagnosis of dementia, and therefore the temporal diagnosis of DLB.

In our study, 67% of DLB patient and 43% of PDD patients reported symptoms suggestive of REM sleep behavior disorder, with no significant difference between the groups. These ratios are comparable with a previous study, which reported 74% in DLB and 58% of non-demented Parkinson’s patients (39). To the best of our knowledge, REM sleep behavior disorder rates in DLB and PDD patients have not previously been directly compared (40, 41).

There are pathological criteria for Parkinson’s disease and DLB, with the pattern of brainstem, limbic and neocortical Lewy bodies being a key factor (2, 42). Revisions to the diagnostic criteria for DLB have improved sensitivity and specificity of clinical diagnosis since the original 1996 criteria, with one recent study showing sensitivity of 70% and specificity >90% for diagnosing DLB using the 2005 consensus criteria (42, 43). While some studies show an association between cognitive decline and cortical synuclein aggregation, the complete picture is more complex, potentially including synergistic association with other aggregated proteins (44). However, to the best of our knowledge, no study has correlated tissue-level diagnosis with detailed neuropsychological profiles or the relative timing of onset of symptoms. Future studies might consent patients at the time of diagnosis, and follow their neuropsychological profile until the time of autopsy.

Importantly, in our study, we found that the interval between the diagnosis of parkinsonism and dementia did not fall into two well-defined categories. This is a major difference compared with previous studies, which by design excluded intermediate cases. For example, Aarsland et al. (5) strictly limited PDD cases to those with tremor and levodopa responsivity and DLB to patients with known postmortem diagnosis. In their study, the mean interval between PD and PDD diagnosis was 12 years. This strategy leaves out intermediate cases that represent the spectrum of the disease and common patients seen in clinic. Narrowly defined studies help increase sensitivity, but may not be generalizable. The average CMI in our study in the PDD population was 7.9 years, ranging from 1.06 to 23 years. Future studies at alternate sites, for example community clinics, could help determine if this spectrum of CMIs also exists outside of a tertiary referral center.

A significant limitation in our study is the fact that both PDD and DLB are diagnosed purely clinically based on retrospective chart review. Beta-amyloid is often found concomitant with alpha-synuclein, with different degrees of overlap increasing clinical uncertainty (2). While pathological and other ancillary data might help clarify our diagnostic effort, our data strongly indicate clinical and neuropsychological data are not enough to distinguish between groups and suggest that temporal onset alone is not sufficient for a clinician to predict and counsel on future symptoms, such as risk of hallucinations or a particular cognitive profile.

Another key limitation is sample size. These patients represent all of the patients who met criteria at the University of Iowa, a major medical center in the central United States, and had high-quality neuropsychological testing. Sample size might be increased by expanding the number of sites. Increasing sample size and power could potentially bring out small differences, but the overall similar cognitive profiles between these groups would make this effort challenging and unlikely to be clinically useful. We are also limited in our ability to precisely identify motor symptom and dementia onset, as patients or families often have motor or cognitive complaints years before the patient presents to clinic. Future studies might address this issue prospectively with objective metrics to study if relative cognitive and motor onset clarifies any differences between PDD and DLB. These studies might also use more targeted neuropsychological tests on inhibition, reasoning, or planning, and other assessments of mood such as delusions, or impulse-control disorders. Furthermore, studying the correlation between CMI and ancillary tests, such as MRI, PET, or CSF studies, may improve understanding of why some patients have Parkinson’s disease for years before onset of cognitive symptoms. Clarifying this issue has clinical relevance as some medications are approved for one disease or the other. Understanding whether these diseases are distinct, or in fact, exist as a spectrum is essential for patients and their families, as well as future research.

## Ethics Statement

This study was carried out in accordance with the recommendations of the University of Iowa Institutional Review Board.

## Author Contributions

GA collected and analyzed data, wrote the manuscript, and helped design the study. AB helped design the study and collected data. ND designed the study, collected and analyzed data, and edited the manuscript. NN helped design the study, analyzed data, and edited the manuscript.

## Conflict of Interest Statement

The authors declare that the research was conducted in the absence of any commercial or financial relationships that could be construed as a potential conflict of interest.

## References

[1] Vann Jones SA O’Brien JT. The prevalence and incidence of dementia with Lewy bodies: a systematic review of population and clinical studies. Psychol Med (2014) 44:673–83. 10.1017/S0033291713000494 23521899

[2] McKeith IG Boeve BF Dickson DW Halliday G Taylor JP Weintraub D Diagnosis and management of dementia with Lewy bodies: fourth consensus report of the DLB Consortium. Neurology (2017) 89:88–100. 10.1212/WNL.0000000000004058 28592453 PMC5496518

[3] Gnanalingham KK Byrne EJ Thornton A Sambrook MA Bannister P. Motor and cognitive function in Lewy body dementia: comparison with Alzheimer’s and Parkinson’s diseases. J Neurol Neurosurg Psychiatry (1997) 62:243–52. 10.1136/jnnp.62.3.243 9069479 PMC1064153

[4] Ballard CG Ayre G O’brien J Sahgal A Mckeith IG Ince PG Simple standardised neuropsychological assessments aid in the differential diagnosis of dementia with Lewy bodies from Alzheimer’s disease and vascular dementia. Dement Geriatr Cogn Disord (1999) 10:104–8. 10.1159/000017109 10026383

[5] Aarsland D Litvan I Salmon D Galasko D Wentzel-Larsen T Larsen JP. Performance on the dementia rating scale in Parkinson’s disease with dementia and dementia with Lewy bodies: comparison with progressive supranuclear palsy and Alzheimer’s disease. J Neurol Neurosurg Psychiatry (2003) 74:1215–20. 10.1136/jnnp.74.9.1215 12933921 PMC1738667

[6] Emre M Aarsland D Brown R Burn DJ Duyckaerts C Mizuno Y Clinical diagnostic criteria for dementia associated with Parkinson’s disease. Mov Disord (2007) 22:1689–707. 10.1002/mds.21507 quiz 1837, 17542011

[7] Hely MA Reid WG Adena MA Halliday GM Morris JG. The Sydney multicenter study of Parkinson’s disease: the inevitability of dementia at 20 years. Mov Disord (2008) 23:837–44. 10.1002/mds.21956 18307261

[8] Fields JA Cognitive and neuropsychiatric features in Parkinson’s and Lewy body dementias. Arch Clin Neuropsychol (2017) 32(7):786–801. 10.1093/arclin/acx085 28961866

[9] Wechsler D Wechsler Adult Intelligence Scale-Third Edition (WAIS–III). San Antonio, TX: Psychological Corporation (1997).

[10] Benton AL A visual retention test for clinical use. Arch NeurPsych. (1945) 54(3):212–16. 10.1001/archneurpsyc.1945.02300090051008 21004267

[11] Benton AL Hamsher DSK Sivan AB Multilingual Aphasia Examination. 2nd ed Iowa City, IA: AJA Associates (1983).

[12] Benton AL Hamsher K Varney N Spreen O Contributions to Neuropsychological Assessment: A Clinical Manual. New York: Oxford University Press (1983).

[13] Schmidt M Rey Auditory and Verbal Learning Test. A Handbook. Los Angeles: Western Psychological Association (1996).

[14] Wechsler D Manual for the Wechsler Memory Scale – Revised. San Antonio, TX: The Psychological Corporation (1987).

[15] Osterrieth PA The challenge of copying a complex figure. Arch Psychol (1944) 30:205–353.

[16] Kaplan E Goodglass H Weintraub S Boston Naming Test. Austin: Pro-ed (2001).

[17] Reitan RM Validity of the trail making test as an indicator of organic brain damage. Percept Mot Skills (1958) 8:271–6. 10.2466/pms.1958.8.3.271

[18] Beck AT Steer RA Brown GK Beck Depression Inventory-II. (Vol. 78). San Antonio: Pearson (1996). p. 490–8.

[19] Yesavage JA Brink TL Rose TL Lum O Huang V Adey M Development and validation of a geriatric depression screening scale: a preliminary report. J Psychiatr Res (1982) 17:37–49. 10.1016/0022-3956(82)90033-4 7183759

[20] Berger JO Pericchi LR Training samples in objective Bayesian model selection. Ann Stat (2004) 32:841–69. 10.1214/009053604000000229

[21] Downes JJ Priestley NM Doran M Ferran J Ghadiali E Cooper P. Intellectual, mnemonic, and frontal functions in dementia with Lewy bodies: a comparison with early and advanced Parkinson’s disease. Behav Neurol (1999) 11:173–83. 10.1155/1999/851860 22387597

[22] Mosimann UP Mather G Wesnes KA O’brien JT Burn DJ Mckeith IG. Visual perception in Parkinson disease dementia and dementia with Lewy bodies. Neurology (2004) 63:2091–6. 10.1212/01.WNL.0000145764.70698.4E 15596755

[23] Bronnick K Cognitive profile in Parkinson’s disease dementia. In: Emre M, editor. Cognitive Impairment and Dementia in Parkinson’s Disease. Oxford: Oxford University Press (2015). p. 27–45.

[24] Filoteo JV Salmon DP Schiehser DM Kane AE Hamilton JM Rilling LM Verbal learning and memory in patients with dementia with Lewy bodies or Parkinson’s disease with dementia. J Clin Exp Neuropsychol (2009) 31:823–34. 10.1080/13803390802572401 19221922 PMC2935683

[25] Mondon K Gochard A Marque A Armand A Beauchamp D Prunier C Visual recognition memory differentiates dementia with Lewy bodies and Parkinson’s disease dementia. J Neurol Neurosurg Psychiatry (2007) 78:738–41. 10.1136/jnnp.2006.104257 17287240 PMC2117680

[26] Janvin CC Larsen JP Salmon DP Galasko D Hugdahl K Aarsland D Cognitive profiles of individual patients with Parkinson’s disease and dementia: comparison with dementia with Lewy bodies and Alzheimer’s disease. Mov Disord (2006) 21:337–42. 10.1002/mds.20726 16211595

[27] Glatt SL Hubble JP Lyons K Paolo A Troster AI Hassanein RE Risk factors for dementia in Parkinson’s disease: effect of education. Neuroepidemiology (1996) 15:20–5. 10.1159/000109885 8719045

[28] Narayanan NS Rodnitzky RL Uc EY. Prefrontal dopamine signaling and cognitive symptoms of Parkinson’s disease. Rev Neurosci (2013) 24:267–78. 10.1515/revneuro-2013-0004 23729617 PMC3836593

[29] Parker KL Ruggiero RN Narayanan NS. Infusion of D1 dopamine receptor agonist into medial frontal cortex disrupts neural correlates of interval timing. Front Behav Neurosci (2015) 9:294. 10.3389/fnbeh.2015.00294 26617499 PMC4639709

[30] Zhang Q Kim YC Narayanan NS. Disease-modifying therapeutic directions for Lewy-Body dementias. Front Neurosci (2015) 9:293. 10.3389/fnins.2015.00293 26347604 PMC4542461

[31] Chen KH Okerstrom KL Kingyon JR Anderson SW Cavanagh JF Narayanan NS. Startle habituation and midfrontal theta activity in Parkinson disease. J Cogn Neurosci (2016) 28:1923–32. 10.1162/jocn_a_01012 27417205 PMC6686857

[32] Wenning GK Scherfler C Granata R Bosch S Verny M Chaudhuri KR Time course of symptomatic orthostatic hypotension and urinary incontinence in patients with postmortem confirmed parkinsonian syndromes: a clinicopathological study. J Neurol Neurosurg Psychiatry (1999) 67:620–3. 10.1136/jnnp.67.5.620 10519868 PMC1736638

[33] Aarsland D Ballard C Mckeith I Perry RH Larsen JP Comparison of extrapyramidal signs in dementia with Lewy bodies and Parkinson’s disease. J Neuropsychiatry Clin Neurosci (2001) 13:374–9. 10.1176/jnp.13.3.374 11514644

[34] Aarsland D Perry R Larsen JP Mckeith IG O’brien JT Perry EK Neuroleptic sensitivity in Parkinson’s disease and parkinsonian dementias. J Clin Psychiatry (2005) 66:633–7. 10.4088/JCP.v66n0514 15889951

[35] Aarsland D Ballard C Larsen JP Mckeith I A comparative study of psychiatric symptoms in dementia with Lewy bodies and Parkinson’s disease with and without dementia. Int J Geriatr Psychiatry (2001) 16:528–36. 10.1002/gps.389 11376470

[36] Ballard CG Aarsland D Mckeith I O’brien J Gray A Cormack F Fluctuations in attention: PD dementia vs DLB with parkinsonism. Neurology (2002) 59:1714–20. 10.1212/01.WNL.0000036908.39696.FD 12473758

[37] Molloy S Mckeith IG O’brien JT Burn DJ. The role of levodopa in the management of dementia with Lewy bodies. J Neurol Neurosurg Psychiatry (2005) 76:1200–3. 10.1136/jnnp.2004.052332 16107351 PMC1739807

[38] Lucetti C Logi C Del Dotto P Berti C Ceravolo R Baldacci F Levodopa response in dementia with Lewy bodies: a 1-year follow-up study. Parkinsonism Relat Disord (2010) 16:522–6. 10.1016/j.parkreldis.2010.06.004 20615745

[39] Munhoz RP Teive HA. REM sleep behaviour disorder: how useful is it for the differential diagnosis of parkinsonism? Clin Neurol Neurosurg (2014) 127:71–4. 10.1016/j.clineuro.2014.09.014 25459246

[40] Boeve BF Silber MH Ferman TJ Kokmen E Smith GE Ivnik RJ REM sleep behavior disorder and degenerative dementia: an association likely reflecting Lewy body disease. Neurology (1998) 51:363–70. 10.1212/WNL.51.2.363 9710004

[41] Pillai JA Leverenz JB Sleep and neurodegeneration: a critical appraisal. Chest (2017) 151(6):1375–86. 10.1016/j.chest.2017.01.002 28087304

[42] McKeith IG Dickson DW Lowe J Emre M O’brien JT Feldman H Diagnosis and management of dementia with Lewy bodies: third report of the DLB Consortium. Neurology (2005) 65:1863–72. 10.1212/01.wnl.0000187889.17253.b1 16237129

[43] Skogseth R Hortobagyi T Soennesyn H Chwiszczuk L Ffytche D Rongve A Accuracy of clinical diagnosis of dementia with Lewy bodies versus neuropathology. J Alzheimers Dis (2017) 59:1139–52. 10.3233/JAD-170274 28731443

[44] Weil RS Lashley TL Bras J Schrag AE Schott JM. Current concepts and controversies in the pathogenesis of Parkinson’s disease dementia and dementia with Lewy bodies. F1000Res (2017) 6:1604. 10.12688/f1000research.11725.1 28928962 PMC5580419

